# Chitosan-Silica Hybrid Biomaterials for Bone Tissue Engineering: A Comparative Study of Xerogels and Aerogels

**DOI:** 10.3390/gels9050383

**Published:** 2023-05-05

**Authors:** Antonio Pérez-Moreno, Manuel Piñero, Rafael Fernández-Montesinos, Gonzalo Pinaglia-Tobaruela, María V. Reyes-Peces, María del Mar Mesa-Díaz, José Ignacio Vilches-Pérez, Luis Esquivias, Nicolás de la Rosa-Fox, Mercedes Salido

**Affiliations:** 1 Departamento de Física de la Materia Condensada, Facultad de Ciencias, Universidad de Cádiz, 11510 Puerto Real, Spain; antoniopmoreno@gmail.com (A.P.-M.); maria.reyes@uca.es (M.V.R.-P.); nicolas.rosafox@uca.es (N.d.l.R.-F.); 2 Instituto de Microscopía Electrónica y Materiales (IMEYMAT), Universidad de Cadiz, 11510 Cádiz, Spain; mariadelmar.mesa@uca.es; 3 Instituto de Biomedicina de Cádiz (INIBICA), Universidad de Cadiz, 11510 Cádiz, Spain; rafafdezmontesinos@gmail.com (R.F.-M.); gonzalo.pinaglia@uca.es (G.P.-T.); ignacio.vilches@uca.es (J.I.V.-P.); 4 Departamento de Histología, SCIBM, Facultad de Medicina, Universidad de Cádiz, 11004 Cádiz, Spain; 5 Departamento de Ingeniería Química, Facultad de Ciencias, Universidad de Cádiz, 11510 Puerto Real, Spain; 6 Departamento de Física de la Materia Condensada, Universidad de Sevilla, 41012 Sevilla, Spain; luisesquivias@us.es

**Keywords:** xerogel, aerogel, chitosan, bioactivity, osteoconduction, focal adhesion, mechanotransduction, bone healing

## Abstract

Chitosan (CS) is a natural biopolymer that shows promise as a biomaterial for bone-tissue regeneration. However, because of their limited ability to induce cell differentiation and high degradation rate, among other drawbacks associated with its use, the creation of CS-based biomaterials remains a problem in bone tissue engineering research. Here we aimed to reduce these disadvantages while retaining the benefits of potential CS biomaterial by combining it with silica to provide sufficient additional structural support for bone regeneration. In this work, CS-silica xerogel and aerogel hybrids with 8 wt.% CS content, designated SCS8X and SCS8A, respectively, were prepared by sol-gel method, either by direct solvent evaporation at the atmospheric pressure or by supercritical drying in CO_2_, respectively. As reported in previous studies, it was confirmed that both types of mesoporous materials exhibited large surface areas (821 m^2^g^−1^–858 m^2^g^−1^) and outstanding bioactivity, as well as osteoconductive properties. In addition to silica and chitosan, the inclusion of 10 wt.% of tricalcium phosphate (TCP), designated SCS8T10X, was also considered, which stimulates a fast bioactive response of the xerogel surface. The results here obtained also demonstrate that xerogels induced earlier cell differentiation than the aerogels with identical composition. In conclusion, our study shows that the sol-gel synthesis of CS-silica xerogels and aerogels enhances not only their bioactive response, but also osteoconduction and cell differentiation properties. Therefore, these new biomaterials should provide adequate secretion of the osteoid for a fast bone regeneration.

## 1. Introduction

Natural polymers and composites for bone-tissue engineering (BTE) have gained considerable interest in recent years due to their good biocompatibility, biodegradability, and ability to mimic the bone extracellular matrix (ECM) [1,2,3,4]. Indeed, by combining biopolymers such as chitosan [5,6], collagen [7], alginate [8], silk fibroin [9], polycaprolactone [10], and gelatin [11,12] with inorganic materials, such as hydroxyapatite [13,14], other types of calcium phosphates [15], or silica hydrogels [16] and aerogels [17,18], hybrid composites with new material properties and applications in various areas of the biomedical field are made possible [4,19,20]. Several investigations on chitosan-based composites for BTE have recently been conducted in order to better understand the function of these novel biomaterials in overcoming the drawbacks of traditional bone-graft treatment. As a result, advancements in chitosan-based scaffolds have produced effective and efficient biological properties through material structural design, primarily through chitosan modification to address its drawbacks, such as poor mechanical properties, bad solubility in water, blood incompatibility and fast biodegradation in the body [21]. For example, CS-based hydrogels have been modified primarily through their main functional groups (OH and NH_2_) with the addition of other compounds or biomaterials to achieve not only enhanced mechanical and physical properties through synergistic effects, but also to improve their biological behavior as bone-regeneration biomaterials. The result is that a lot of CS-based materials have been investigated and described in the literature. On this premise, hydrogel nanocomposites consisting of CS and polyhedral oligomeric silsesquioxanes (POSS) have demonstrated excellent in vitro biocompatibility and drug-release capabilities [22]. Additionally, novel hybrid organic–inorganic porous scaffolds with an interconnected pore network, excellent swelling properties, and adjustable degradation rate have been created from chitosan nanofibers modified with silane coupling agents and polyvinyl alcohol (PVA) [23]. In vitro cell culture investigations have also proven its cytocompatibility and prospective application in BTE.

Among these hybrid materials, the chitosan-silica system has received a lot of attention because of its outstanding bone regeneration characteristics, including osteoconductive and drug transport ability, which allows for the regulated release of pharmaceuticals such as growth factors or antibiotics [5,24,25,26]. As a result, several experiments using chitosan and inorganic silica gels—both with and without chemical alteration of its molecular structure—have been published. In both situations, CS has been demonstrated to be advantageous for BTE, regardless of the drying method (evaporative or supercritical drying) used to create porous CS-silica biomaterials for xerogel or aerogel [27,28,29,30]. The resulting specimens have demonstrated great mechanical strength, making them suitable for load-bearing applications. These hybrid biomaterials also have additional advantages for bone tissue repairing, such as increased porosity and specific surface area, which improve cell adhesion and proliferation [16,31].

To date, many methods such as the sol-gel process [32,33,34], electrospinning [35], spray drying [36], and freeze or supercritical drying [37,38], have been used to produce CS-silica biomaterials with varying microstructure and morphology. Among the numerous methods currently being utilized, the sol-gel method has been extensively used to produce a wide range of organic–inorganic hybrid materials due to its unique benefits, such as chemical homogeneity and purity, as well as the ability to regulate material structure at the nanoscale. So far, silane coupling agents such as aminopropyltriethoxysilane (APTES) or 3-glycidoxypropyltrimethoxysilane (GPTMS), have been used to crosslink the silica and the polymer organic network of chitosan to create mechanically strengthened hybrid composites via sol-gel [28,39]. However, studies have shown that these substances can be hydrolyzed in aqueous solutions, including bodily fluids. Depending on the concentration, this could result in the creation of cytotoxic degradation [40,41]. Incorporating calcium phosphates (CaP) into hybrid biomaterials, on the other hand, can enhance cell compatibility and encourage cell adhesion and proliferation, making these hybrids more appropriate for bone regeneration uses [1,42,43]. Various mesoporous CS–silica hybrids incorporating CaP have been prepared by sol-gel, including xerogels (dried by conventional evaporation of solvents) [27,33] and aerogels (dried in supercritical CO_2_) [28] until now. Obviously, the textural properties of these hybrids are dependent on the drying processes performed. Indeed, they present large surface areas and also have a high potential as support matrices for replicating the structure of the native extracellular matrix (ECM) [27,29].

We are interested in these CS–SiO_2_ hybrid materials as bone substitutes, because their large surface area provides support for other reactive species. However, we must develop a systematic understanding of how various material substrates may influence the bioactive and biological reactions. Thus, while some information exists about how the CS composition affects the textural properties and osteoconductive role of the final hybrid xerogels [27] and aerogels [29], there is little information about how initial cell differentiation, which is linked to mechanotransduction processes, is affected differently in the presence of these type of biomaterials.

Thus, we have created a CS–silica xerogel and aerogel monoliths using a previously described sol-gel approach [38,39] utilizing TEOS and CS powder as biomaterial precursors. For the study, we selected composition samples with well-known bioactive and osteoconductive characteristics, and we compared their effectiveness as bone-healing materials. In particular, we chose silica hybrid samples with an 8 wt.% CS content and no additional chemical additives or crosslinkers in order to avoid possible cytotoxic side effects. According to our previous results, (Perez-Moreno et al., 2020; Pérez-Moreno et al. 2021) this synthetic procedure guarantees that the resulting biomaterials will present high in vitro bioactivity response as well as osteoconduction behavior in cell culture media [27,29] Additionally, a 10 wt.% TCP was added prior to gelation to the original 8 wt.% CS–silica sol in order to evaluate the effects of CaP on cellular development. Because TCP leaching was observed during the CO_2_ supercritical fluid extraction technique to produce the corresponding aerogels, the incorporation of TCP was only taken into account for samples in the xerogel stage. The resulting samples were characterized by thermogravimetric analysis (TGA) and Fourier transform infrared spectroscopy (FTIR). The microstructure and bioactivity were assessed by N_2_-physisorption analysis and SEM, and correlated with actin cytoskeletal changes and focal adhesion maturation in order to identify the best platform for bone healing.

## 2. Results and Discussion

Our goal in this comparative research was to examine the similarities and differences between xerogels and aerogels based on the silica/chitosan hybrid system, with a focus on its use in bone regeneration. This was undertaken from the viewpoint of prior findings [27,29] taking into account the incorporation of TCP in the setting of xerogels. Prior to conventional evaporative drying, the wet gel samples were washed with ethanol solvent for a week to produce the corresponding xerogels. Alternatively, supercritical CO_2_ was used in the drying process to produce the equivalent aerogels. The samples used in this study were chosen based on their in vitro biological performance, as well as well-known bioactive responses in simulated body fluid (SBF), and their synthesis process is discussed in more detail in Section 4.1. Aerogel samples were labeled SCS8A (aerogel), SCS8X (xerogel), and SCS8T10X (a xerogel containing 10 wt.% TCP). In addition, xerogel and aerogels of pure silica (SiO_2_X and SiO_2_A, respectively) were synthesized and included in this study as references. According to earlier research, this variety of compositions offers the materials with enhanced bioactivity as well as the necessary hybrid network stability to avoid undesirable fast biodegradation [27,29]. However, small differences between their biochemical and biophysical responses when exposed to simulated body fluid (SBF) and cell culture, can allow for the best performance of these types of biomaterials to be identified.

### 2.1. Physical and Textural Properties of Xerogels and Aerogels

**Figure 1:**
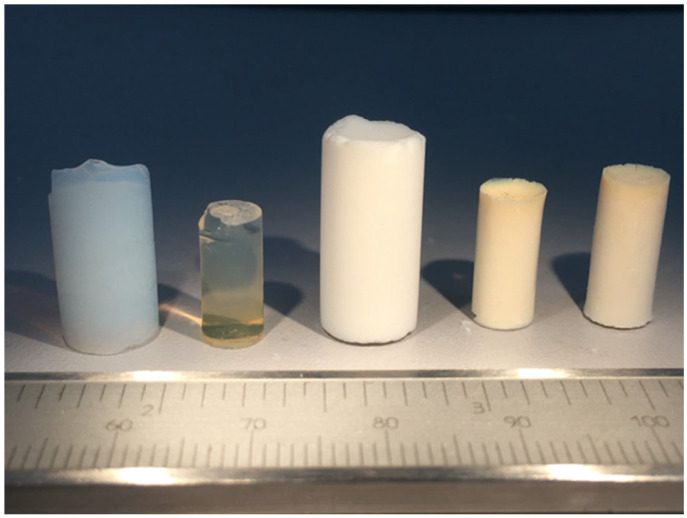
Representative sample set of the studied samples. From left to right: SiO_2_A, SiO_2_X, SCS8A, SCS8X and SCS8T10X.

Table 1 displays the physical properties of the samples under consideration. In all cases, homogeneous and elastic samples in the shape of monolithic cylinders were obtained, with bulk densities ranging from 0.18–0.19 gcm^−3^ in the case of aerogels and from 0.49 to 0.61 gcm^−3^ in xerogels. A significant volume shrinkage of up to 30–33% in aerogels and higher in xerogels (67–76%) was also observed (see Figure 1). Given the high hydrolysis ratio used in the synthesis (Rw = 30), these extremely large shrinkage values were attributed to both drying processes and conventional post-processing [44,45], including polycondensation continuity of the hybrid networks during washing and aging periods, an outcome which was particularly manifest in the samples undergoing solvent evaporation.

Additionally, the textural parameters obtained from N_2_-physisorption experiments are shown in Table 1 for the corresponding hybrid materials (SCS8A, SCS8X and SCS8T10X), and their respective pure silica matrices (SiO_2_A and SiO_2_X). In general, the xerogel samples showed rather high specific surface areas, varying from approximately 734 to 821 m^2^g^−1^ for SCS8T10X and SCS8X samples, respectively. They also exhibited relatively low pore volumes of about 1.0 cm^3^g^−1^ for both the pure silica xerogel and SCS8X sample. This volume increased by 50% when 10 wt.% TCP was incorporated with the SCS8 sample, allowing a xerogel sample with 1.5 gcm^−3^ of pore volume and also the highest pore size (7.5 nm) of all the three xerogel samples involved.

The before-mentioned results should be related to the presence of a certain amount of Ca^2+^ in dissolution after the TCP addition, providing the formation of calcium phosphate hydrates and resulting in a composite of interest in biomedical applications, particularly for bone-tissue engineering and repair [46,47]. As observed in Table 1, the xerogels displayed 70–75% smaller pore volumes and pore sizes than comparable aerogels, a result that can be attributed to the different drying process mechanisms involved. All these findings are consistent with aerogels of mesopore interconnected structures and high surface areas ranging from 857 m^2^g^−1^ to approximately 978 m^2^g^−1^ for the SCS8A and SiO_2_A samples.

**Figure 2:**
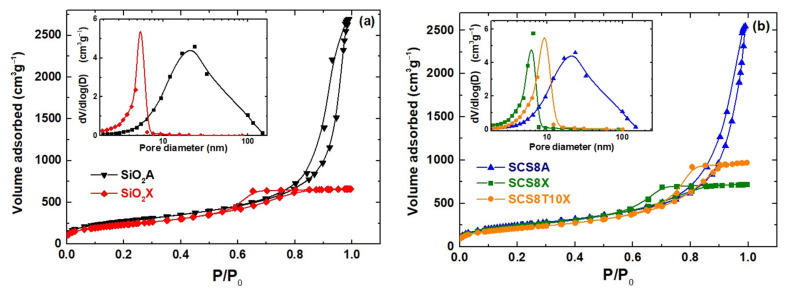
N_2_-isotherms and pore size distribution (see insets) of (**a**) pure silica aerogel (SiO_2_A) and xerogel (SiO_2_X), and (**b**) of the three biomaterials under study (SCS8A, SCS8X and SCS8T10X).

Figure 2a displays the N_2_-physisorption isotherms for the pure silica aerogel and the xerogel samples, revealing that the aerogel SiO_2_A adsorbs almost four times the volume of gas at high pressures as xerogel SiO_2_X, which undergoes stabilization at high relative pressures (0.8–1.0). This is reflected by a reduction in surface area from 978.2 to 807.5 m^2^g^−1^ and also pore size from 16.9 to 4.7 nm (Table 1). Additionally, type IV isotherms with H1 hysteresis loops were observed in both cases, indicating the existence of an interconnected mesopore network with widths between 2 and 50 nm [48], generated by the organic and inorganic precursors and the high hydrolysis ratio employed (30:1) [45]. The corresponding N_2_-isotherms of the SCS8A, SCS8X, and SCS8T10X hybrid biomaterials are shown in Figure 2b, exhibiting characteristics similar to those of their respective inorganic matrices. Both SCS8X and SCS8T10X xerogel isotherms presented a well-defined horizontal plateau at relative pressures of 0.8–1.0 and 0.9–1.0, and a sharp step in the desorption branch at P/P0 values of 0.5–0.6 and 0.6–0.7, respectively. The shape of these isotherms and hysteresis loops is characteristic of cylindrical pores with a narrow pore size distribution and high pore size uniformity [49]. The pore size distribution (PSD) (see insets in Figure 2a,b) showed that the pore volume of the xerogels was mostly in the mesopore domain, with diameters in the 2–16 nm range, while its maximum was situated between 5.0–7.5 nm. Moreover, the t-plot analysis revealed the absence of micropores in all xerogel and aerogel samples [50]. Besides this, the isotherm curves of the aerogels have a steep adsorption branch, confirming their large surface area, followed by a large desorption hysteresis loop that closes at a lower relative pressure than the adsorption branch, which indicates a broad range of pore sizes from approximately 6 to 130 nm, noticeably invading the macropore region, but with the most abundant value of around 17 nm, in the mesopore domain (see Figure 2a,b inset). A cylindrical-like pore geometry with small openings was also suggested by the loop shape in this case, which could be caused by aggregates or bundles of cylindrical pores or interconnected channels [51].

### 2.2. Thermogravimetric Analysis

**Figure 3:**
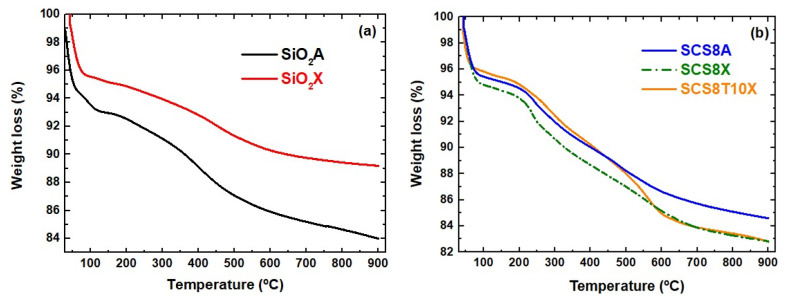
Thermograms of (**a**) (SiO_2_A) and (SiO_2_X) pure silica samples, and (**b**) of SCS8A, SCS8X, and SCS8T10X biomaterials.

The thermal stability and decomposition behavior of the different materials was examined by thermogravimetric analysis. Figure 3a shows the thermogram curves for SiO_2_A and SiO_2_X silica matrices, while Figure 3b shows the thermogram curves for SCS8A, SCS8X, and SCS8T10X hybrid biomaterials. An initial weight loss in the range of 50–150 °C, accounting for the loss of adsorbed moisture, was observed in all cases (Figure 3a,b), suggesting the typical hydrophilic nature of all surfaces, most notably in the case of the SiO_2_A aerogel specimen. The second weight loss occurred in the temperature range 150 °C–450 °C (Figure 3a). This is because of elimination of water molecules, which are produced by the dehydration of the silica network that develops during the hydrolysis and condensation of TEOS, with the silica aerogel losing additional weight. The degradation of any residual organic component contained in the silica aerogel caused a third weight loss that occurred between 450 and 600 °C (Figure 3a). This was mostly due to the decomposition of ethanol and unreacted precursors. Finally, the dehydroxylation of the silica network causes the fourth weight loss, which occurs between 600 and 900 °C, which is attributed to the elimination of hydroxyl groups from the silica network, which causes the creation of a more organized and denser silica structure [29]. Figure 3b shows comparable thermal processes for the SCS8A aerogel and SCS8X and SCS8T10 xerogel samples, including typical weight losses between 150 and 350 °C and 350–600 °C related to the degradation of chitosan [27,52]. In addition, a notable weight loss was observed in the thermogram of SCS8T10X between 350 and 600 °C, which was caused by the decomposition of TCP contained in the hybrid xerogel [27,53].

### 2.3. FTIR Spectral Analysis

**Figure 4:**
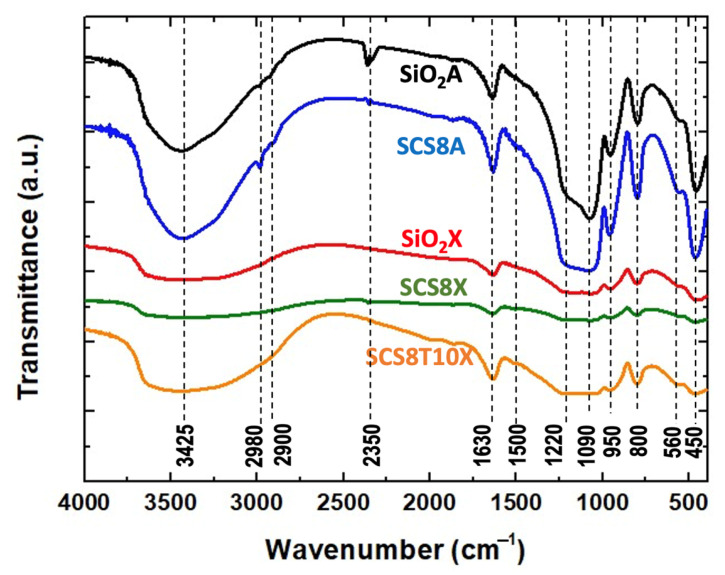
FTIR spectra of aerogel and xerogel pure and hybrid samples containing 8 wt.% chitosan and 10 wt.% TCP.

A few significant differences were observed in the FTIR spectra of the aerogels and the xerogels (Figure 4). First, aerogels (SiO_2_S and SCS8A) often display larger and more intense bands at 450 cm^−1^ and 800 cm^−1^ (Si-O-Si bending vibration), and in the 1050–1200 cm^−1^ range (Si-O-Si stretching vibration), which are characteristics of the silica network [54]. This is because the supercritical drying technique used to create aerogels results in a more densely cross-linked network structure with a greater degree of polymerization, and therefore a more compact pore network structure. A shorter and narrower band, which denotes a lower level of polymerization and a more porous network structure, is generally observed in the same frequency region for the xerogels (SiO_2_X, SCS8X, and SCS8T10X). The stretching vibrations of the OH groups, which correspond to the 3200–3700 cm^−1^ range, where aerogels also show more prominent peaks, suggest a larger number of residual silanol groups (-Si-OH) and adsorbed water owing to their typical hydrophilic character. In addition, the peaks at 950 cm^−1^ and at around 2350 cm^−1^ in the SiO_2_A sample were attributed to the stretching and bending vibrations of the Si-OH bond, respectively, thus confirming the high -OH density at the aerogel surfaces [29]. Peaks at approximately 2900–3000 cm^−1^ were present only in the aerogels (SiO_2_A and SCS8A) accounting for C-H stretching vibrations due to the use of ethanol as s M!”olvent to preserve the samples before supercritical drying [29].

However, the incorporation of chitosan into the hybrid gel structures of SCS8A, SCS8X, and SCS8T10X is difficult to discern, given that the majority of FTIR peaks overlap with those of silica [27]. The presence of chitosan in these hybrids provides the appearance of peaks at approximately 1150 cm^−1^ (stretching vibration of C-O-C), 1400 cm^−1^ (bending vibrations of C-H bond), 1560 cm^−1^, and 1650 cm^−1^ (bending vibrations of the N-H and C=O groups, respectively). Both bands (amide I and amide II) may also be signals of the presence of chitosan. In addition, the broad peak in the range of 3200–3400 cm^−1^ can indicate stretching vibrations of -OH and N-H in chitosan [30].

Additionally, the FTIR spectrum of SCS8T10X includes a broad peak at approximately 1100–1200 cm^−1^, which corresponds to the stretching vibration of the phosphate (PO_4_) groups in TCP, while bending vibrations may appear between 600–900 cm^−1^. Finally, a peak at approximately 400–600 cm^−1^ may be indicative of the stretching vibrations of Ca-O bonds in TCP [27].

### 2.4. In Vitro Bioactivity in SBF

**Figure 5:**
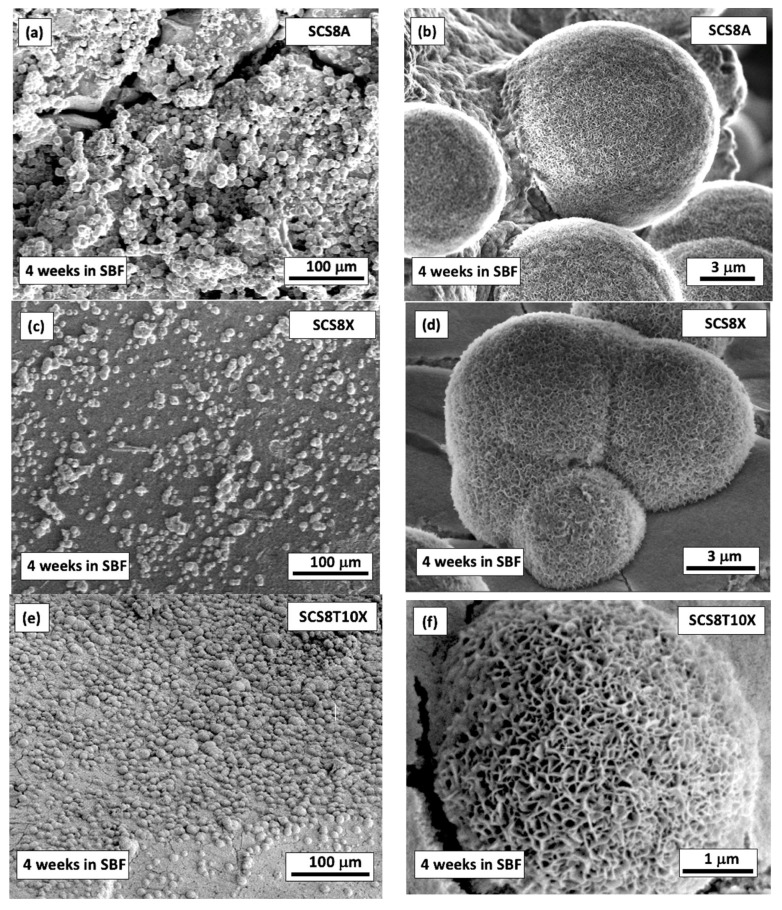
SEM micrographs of the surfaces of (**a**,**b**) SCS8A, (**c**,**d**) SCS8X, and (**e**,**f**) SCS8T10X hybrid gels after immersion in SBF for 28 days at 37 °C displaying strong bioactive response through the formation of a hydroxyapatite (HAp) layer.

Figure 5 shows SEM micrographs of the hybrids after they were tested for bioactivity in SBF. The interaction of calcium and phosphate ions from the surrounding media formed an apatite-like structure on the surfaces of all samples after immersion for 28 days in SBF. As previously shown, the textural characteristics of the gels, along with the composition of the mineralized products, both affected the morphology and particle size of the HAp aggregates deposited on the three different surfaces [11,27,29]. As an illustration, Figure 3a,b show how the aerogel surface of SCS8A enables the formation of HAp spherulitic particles, which develop into an abundant accumulation with a nearly ideal spheric shape of uniform size (~10 μm). Instead, the corresponding xerogel (SCS8X) allows for the formation of HAp spherulites, which appear as agglomerates of varying sizes (~4–8 μm) and cover the surface to a lesser extent than the aerogel (see Figure 3c,d). Finally, as shown in Figure 3e,f, the TCP-containing xerogel sample (SCS8T10X) promoted the formation of an apatite layer that covered almost the entire surface of the substrate and had particle sizes of about 5 μm.

These results show the potential for the proposed biomimetic mineralization method to produce materials recovered by hydroxyapatite layers in all cases, with particle sizes ranging from 5 to 10 mm and a Ca/P ratio that is nearly stoichiometric (1.67) as described in previous work [27,29], with biocompatibility properties, which may be useful for a variety of biomedical applications as will be discussed below.

### 2.5. Osteoblast Behavior

The barrier that exists between a host and any implanted device that forms at the cell–biomaterial interface is considerably more than just a simple border; instead, it provides key cues for cell adhesion, subsequent induction, and tissue neogenesis as described by Biggs et al. [55]. A construct’s function and cytocompatibility can be evaluated in vitro by examining cell viability and adherence at the substratum interface. The initial polarization of HOB cells was evident from 24 h onwards, and then followed by cell-adhesion protein expression and cell modifications identified as early osteoblast differentiation markers, which can be attributed to the biomaterial, as we and others have previously described [28,56,57,58,59,60].

**Figure 6:**
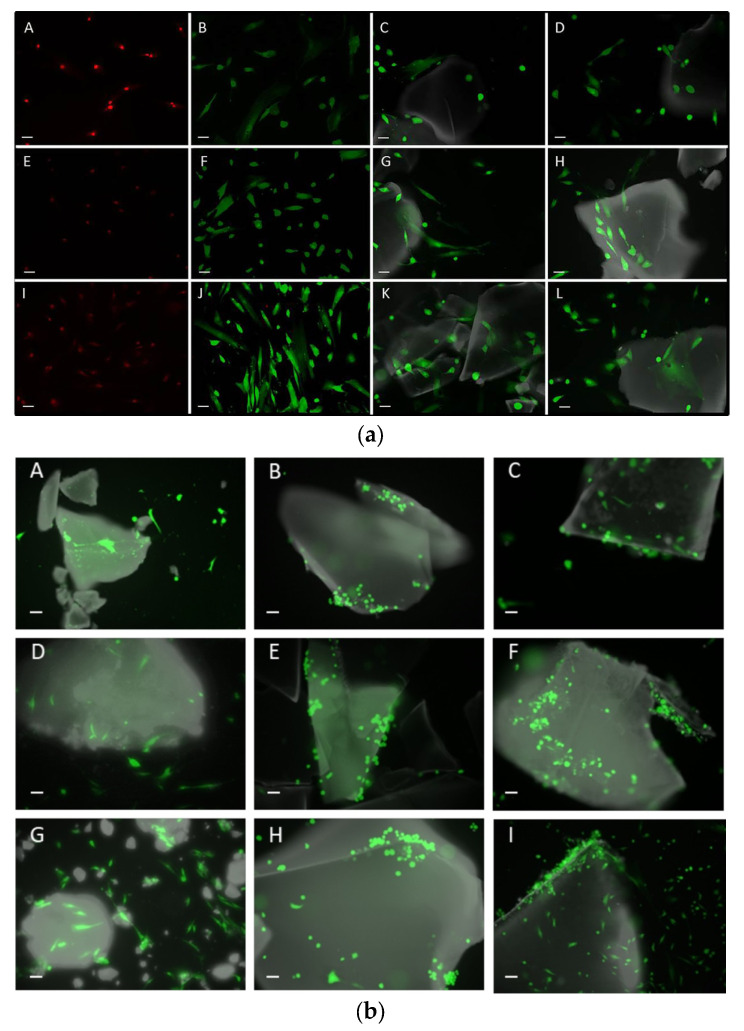
(**a**) Live/dead staining of HOB culture. Negative controls treated with 70% methanol for 30 min prior to staining: (**A**) 48 h HOB cultures, (**E**) 72 h HOB cultures and (**I**) one-week HOB cultures. The cultures of live control osteoblasts (positive control) were untreated cells grown underoptimal conditions at the described timepoints. Positive controls: (**B**) 48 h HOB cultures, (**F**) 72 h HOB cultures and (**J**) one-week HOB cultures. HOB cultures with SiO_2_A: (**C**) 48 h HOB cultures, (**G**) 72 h HOB cultures and (**K**) one-week HOB cultures. HOB cultures with SiO_2_X: (**D**) 48 h HOB cultures, (**H**) 72 h HOB cultures and (**L**) one-week HOB cultures. Green: living cells; red: dead cells and materials in gray. Scale: 20 μm. (**b**) Live/dead staining of HOB cells grown in the presence of SCS8A: (**A**) 48 h HOB cultures, (**D**) 72 h HOB cultures and (**G**) one-week HOB cultures. HOB cultures with SCS8X: (**B**) 48 h HOB cultures, (**E**) 72 h HOB cultures and (**H**) one-week HOB cultures. HOB cultures with SCS8T10X: (**C**) 48 h HOB cultures, (**F**) 72 h HOB cultures and (**I**) one-week HOB cultures. Live cells flouresce green; dead cells are imaged in red and materials in gray. Scale bar: 20 μm.

At seeding, cell viability reached 98%. No appreciable apoptotic events were observed in either the experimental or control groups. The live dead assay was used in experimental groups to evaluate cell growth and viability, with a majority of osteoblasts, at any experimental time, were in a viable state (green), with only a small number of dead cells (red) (see Figure 6a,b).

**Figure 7:**
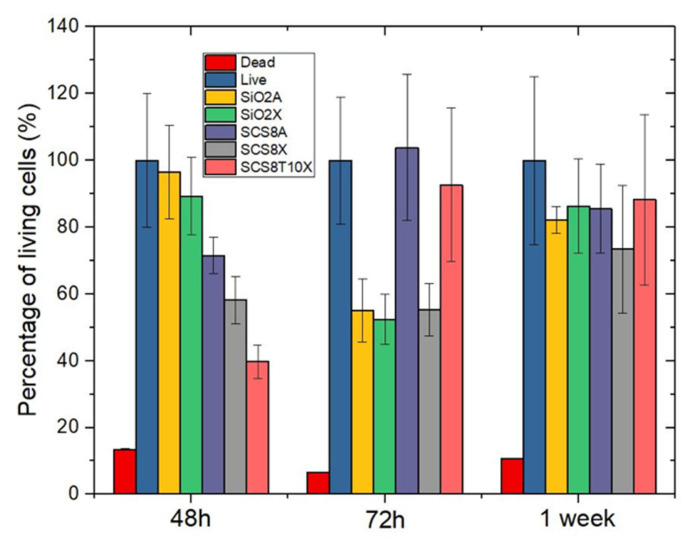
Histogram representations as mean ± SEM.of the percentage of living HOB cells. Negative controls (dead l) were treated for 30 min before labelling with 70% methanol. Live represent the reference value i.e., 100% of living cells obtained from untreated cultures grown under optimal conditions at the experimental times. Quantification analyzed in 10 images per experiment, in triplicate.

In the first 48 and 72 hours of cell cultures, some of the materials cause variations in the cell proliferation of osteoblasts, although after one week of cell culture there is no difference between the materials, even when compared with the positive control. Therefore, the materials do not appear to affect the optimal growth of osteoblasts. (See Figure 7).

### 2.6. Cell Morphology, Cytoskeletal Organization, and Focal Adhesions

The importance and development of biochemical and biophysical features regulating cell–biomaterial interactions in the early stages are of capital importance in orchestrating the complex material–cytoskeleton crosstalk occurring at the interface. Although the precise underlying biomolecular mechanisms are not well defined, there is growing evidence that cytoskeleton-mediated signaling could prove to be sufficient to start and sustain differentiation programs [55,60]. In this milieu, focal adhesions (FAs) have a pivotal function in this process as a mechanical link between the cytoskeleton and the extracellular environment. They change in size, maturation stages, and in distribution according to the forces acting on them, as well as cytoskeleton changes according to FAs spatial distribution and size [61,62,63,64]. Two distinct forms of FAs have been identified that differ depending on cellular motility. In cells that retain their migratory capacity, small FAs are predominant, mostly composed of vinculin and talin, and appear on the leading edges of filopodia or lamellipodia.

These are also called immature punctate nanoscale (0.2 μm^2^) FAs and can undergo rapid turnover within the lamellipodium or mature into larger FAs influenced by cytoskeletal tension and motion state of the cell. Mature FAs are large (1.0–10 mm^2^) and predominantly composed of paxillin, vinculin, focal adhesion kinases and α actinin. During the migration phase they anchor cells, to maintain cellular morphology and tensional homeostasis. In resting and non-migrating osteoblasts, they can be localized or even formed throughout the whole cytoplasm [11,65,66,67].

**Figure 8:**
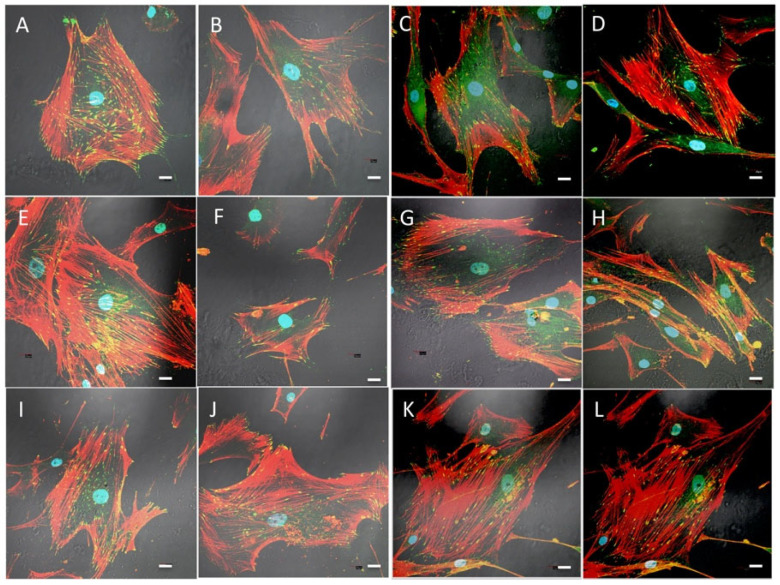
Immunolabelling and confocal examination of actin cytoskeleton with rhodamine phalloidin (red) and vinculin for focal adhesions (green) of HOB^®^ osteoblasts cultured in the presence of: (**A**,**B**) xerogel SCS8X; (**C**,**D**) SCS8T10X, 48 hours; (**E**,**F**) xerogel SCS8X after 72 h,(**G**,**H**) SCS8T10X after 72 h in culture; (**I**,**J**) xerogel SCS8X, 1 week, and (**K**,**L**) SCS8T10X after 1 week in culture. Blue, DAPI-labelled nuclei. Scale bar: 20 μm.

After osteoblast immunolabelling with rhodamine phalloidin and antivinculin antibodies, we observed focal adhesion development and cytoskeletal changes in both the aerogel and xerogel groups. Once the distribution of vinculin-positive events was assessed, we used image analysis to measure the size of FAs in the proposed silica–chitosan biomaterials (Figure 8).

Our findings showed that predominantly small- and medium-sized FAs, as a sign of cell migration, were identified after 48 h in culture in SCS8X and SCS8T10X with accompanying changes in the cytoskeleton. In the silica–chitosan xerogel groups (Figure 8), the development of stress fibers was observed from 48 h, increased with time, and were arranged in the cell periphery and tipped with FAs. After 1 week (Figure 8 I–L), actin stress fibers appear clearly organized on the entire mobile surface of the cell and reinforced by a large number of FAs on the cell’s leading edges

**Figure 9:**
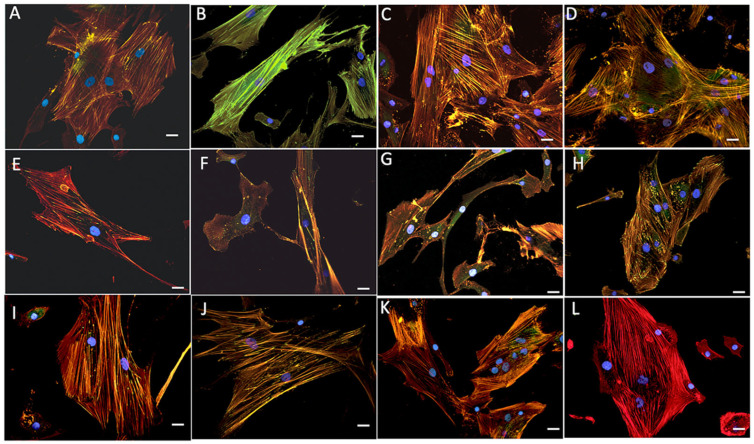
Immunolabelling with rhodamine phalloidin and confocal examination of actin cytoskeleton (red) and vinculin (green) for FAs of HOB^®^ osteoblasts grown in the presence of: (**A**,**B**) SiO_2_A, 48 h culture; (**C**,**D**) SiO_2_X, 48 h culture; (**E**,**F**) SiO_2_A 72 h culture; (**G**,**H**) SiO_2_X 72 h culture; (**I**,**J**) SiO_2_A 1 week culture; (**K**,**L**) SiO_2_X after 1 week in culture. Blue, DAPI-labelled nuclei. Scale bar: 20 μm.

Comparative images of osteoblasts cultured in the presence of SiO_2_X or SiO_2_A samples are shown in Figure 9 and revealed quite a different pattern both for focal adhesion distribution and also for cytoskeletal changes. Scarce and poorly developed FAs appeared in the first 48 and 72 h, and even after one week, especially in the SiO_2_A groups, which can be associated with cells that are still in the migratory phase. In the SiO_2_X groups, a greater differentiation of actin filaments towards stress fibers was observed after 1 week in culture, although at all experimental times, the cellular response was lower than that observed in the hybrid groups.

**Figure 10:**
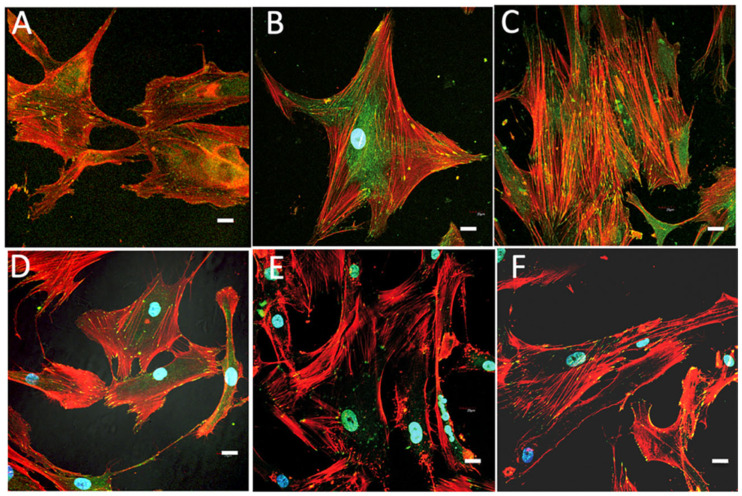
Immunolabelling and confocal examination of actin cytoskeleton with rhodamine phalloidin (red) and vinculin (green) for FAs of HOB^®^ osteoblasts growing in culture in the presence of: SCS8A (**A**), 48 h culture; (**B**) 72 h culture; (**C**) 1 week culture and osteoblasts growing in glass (controls) (**D**), 48 h culture; (**E**) 72 h culture; (**F**) 1 week culture. Blue, DAPI-labelled nuclei. Scale bar: 20 μm.

According to our data and the literature, osteoblasts grown in the presence of SCS8A seem to maintain their migration capability, according to focal adhesion patterns with an elongated morphology over time. In the aerogel groups (Figure 10A–C), the appearance of stress fibers occurred somewhat later, being already evident after 72 h, in which they presented a peripheral distribution associated with lamellipodia and filopodia and were very evident after one week of culture. Finally, in the control groups on glass (Figure 10D–F), elongated and narrow cells with few filopodia, some lamellipodia, few non-polarized FAs, and a cytoskeleton pattern with no obvious stress fibers were observed.

**Figure 11:**
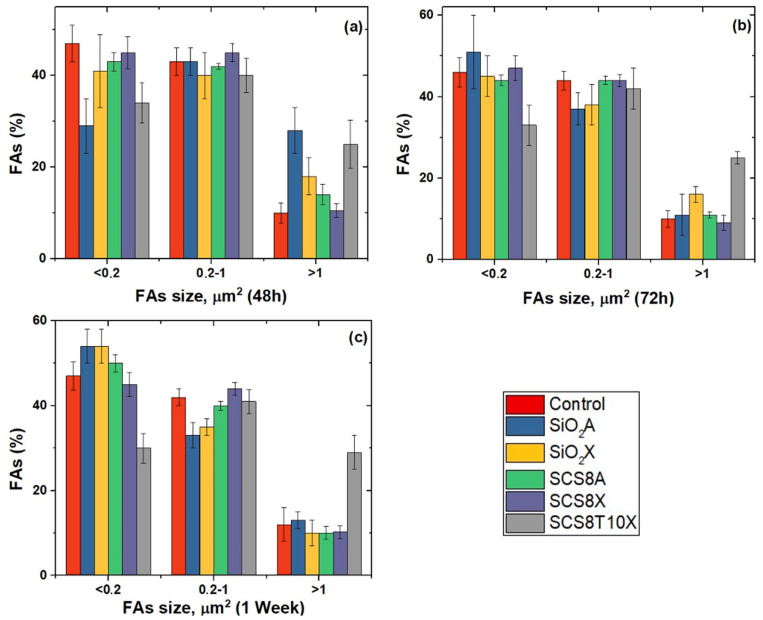
FA size in HOB^®^ cultured on the xerogels. Time-dependent percentage after (**a**) 48 h, (**b**) 72 h, (**c**) 1 week. One way analysis of variance. Statistical significance was defined as *p* < 0.05.

**Figure 12:**
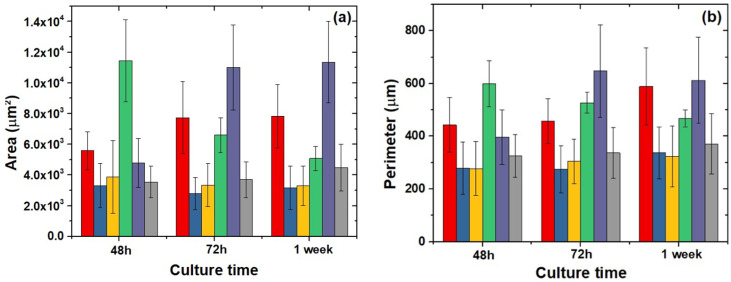

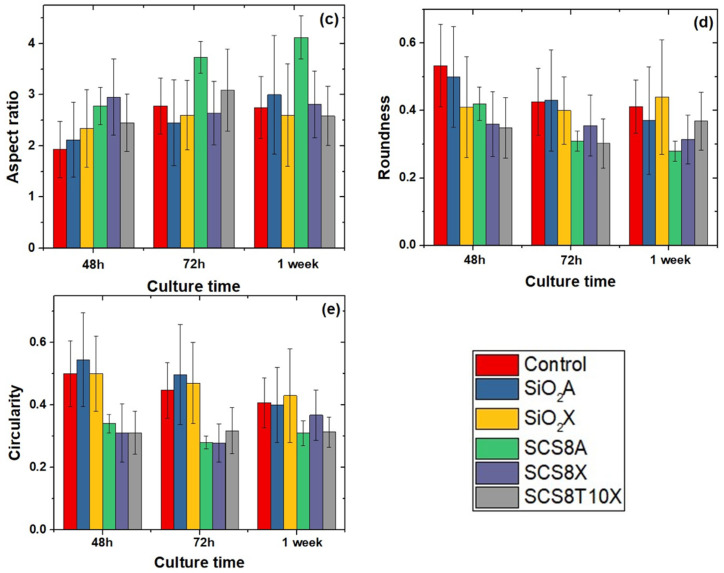
Quantification of shape variables. (**a**) Cell spread area, (**b**) cell aspect ratio, (**c**) circularity, (**d**) cell perimeter, and (**e**) cell roundness. One way ANOVA. Significance: *p* < 0.05.

Image analysis confirmed the data described above, with predominantly small- and medium-sized FAs (Figure 11), as expression of cell migration, after 48 h in culture in SCS8X and SCS8T10X. Concordantly, minor morphological changes were identified in these groups (Figure 12).

In SiO_2_A groups, as well as in SiO_2_X groups, small FAs predominated and the percentage increased after 72 h and 1 week, while medium- and large-sized FAs percentages decreased with time (Figure 11). This tendency persisted for 72 h and 1 week in culture. Nevertheless, in the SCS8T10 X groups, the percentage of mature FAs, sized > 1 square micron, remain stable for 48 h onwards and significantly increased with respect to the rest of the experimental groups after one week of culture. In control cells, small- and medium-sized FAs predominate at any experimental times.

Morphological analysis of shape parameters revealed changes in cell area, mainly in SCS8A groups, which were the largest at initial times and elongated with time, as confirmed by the perimeter and aspect ratio data shown in Figure 12a–c. In contrast, cells grown on SCS8X increased both in area and perimeter, and consequently in aspect ratio, from 48 h to 72 h and finally 1 week. These data were consistent with FA expression, with small- and medium-sized FAs during all experimental times. Osteoblasts grown in the control groups and in biomaterials composed only of SiO_2_ presented a less complex cell morphology with the highest circularity values.

Bone tissue capability of self-regeneration in response to a variety of diseases and injuries is mainly mediated by osteoblasts, cells from the mesenchymal lineage that can divide and generate both collagen fibers and extracellular bone matrix (EBM) components. During both osteogenesis and regeneration processes, osteoblasts differentiate into a second type of bony cell called osteocytes, which are in the final differentiation stage of the osteoblastic lineage. During the process, osteoblast morphology changes, and long branched osteocytic processes emerge from a smaller cell body, while the extracellular matrix osteoid is formed. Osteocytic channels within progressively calcified EBM are then created, while osteocytic processes grow to maintain cells in contact with nearby capillaries, osteocytes, and border cells. Osteocytic processes also play an important role in mechanotransduction events by keeping the cytoskeleton in deep contact with focal adhesion contacts, thus allowing mechanical forces from the surrounding environment to modulate cell differentiation [66,68].

Proteins, primarily serum vitronectin and fibronectin, begin to cling to the biomaterial surface when exposed to a supplemented cell culture medium in vitro. Cell membrane integrins can recognize specific binding sites on these proteins. Following this, via linker proteins such as vinculin, the integrins assemble into what are known as FA complexes and bind to F-actin. Osteogenic cells have a highly developed actin cytoskeleton, and it is well known that mechanical stress in their microenvironment plays a role in determining their development [56,57,58,69].

The role of FAs during initial cell adhesion couples their role as mechanosensors for biophysical and biochemical properties of the material with continuous assembly and disassembly as the cell moves, leading to focal adhesion changes in number and size, as described in the results section. Concordant cytoskeletal changes in actin networks leading to stress fiber development and changes in cell morphology are observed when osteoblasts are in the presence of an optimal scaffold [70].

We previously demonstrated that FAs are involved in the surface recognition process, which leads to cell adhesion and mechanotransduction-induced alterations in osteoblasts. Additional cues for cell placement, spreading, and differentiation can be achieved when the nanoscale biomaterial surface is chemically optimal. As we and others have previously described, chitosan is a natural polymer that offers adequate biocompatibility, biodegradability, hydrophilicity, and nontoxicity.

## 3. Conclusions

Nanoscale chitosan-based biomaterial surfaces described in the paper offer an adequate set of characteristics for BTE. In addition, they are chemically attractive and provide additional cues for cell positioning, spreading, and differentiation. The best osteoblastic response was observed in the presence of SCS8T10X with indicators that would hopefully favor adequate secretion of osteoid. SCS8A and SCS8X samples required more time to induce an osteoblastic differentiation response comparable to that of TCP-enriched samples, and osteoblasts grown on these samples retained a good migration capacity for quite some time. All xerogel samples appeared to induce cell differentiation earlier than the aerogels with identical compositions.

Finally, the data obtained for the samples composed only of SiO_2_ showed the least influence on the differentiation indicators used in the time periods studied, and the cellular response was much slower. We can conclude that nanoscale chitosan-based biomaterial surfaces offer an adequate set of characteristics, such as biocompatibility, biodegradability, hydrophilicity, and non-toxicity. In addition, they are chemically appealing, providing additional cues for cell placement, osteoblast spreading, and cell differentiation, which can be improved by the addition of chitosan, TCP, or both. Therefore, the adequacy of the different parameters influencing synthesis (including drying strategies) must be considered to optimize the osteoblastic response.

## 4. Materials and Methods

### 4.1. Materials

Chitosan (CS; 50,000–190,000 Da; 75–85% deacetylation degree) was provided by Sigma Aldrich (St. Louis, MI, USA). Tetraethyl-orthosilicate (TEOS, 99%) and hydrochloric acid (37%, Pharma grade) were supplied for Alfa Aesar (Haverhill, MA, USA). Tri-calcium phosphate (TCP, pure, pharma grade) and absolute ethanol (99.5%) were obtained from Panreac (Barcelona, Spain), HOB^®^ human osteoblasts, fetal calf serum, and osteoblast growing medium (Promocell, Heidelberg, Germany) paraformaldehyde, PBS, Triton x-100, bovine serum albumin, methanol, rhodamine phalloidin, and monoclonal anti-vinculin FITC conjugate were all purchased from Sigma Aldrich, (St. Louis, MI, USA) along with Hard Set Vectashield with DAPI ^®^ (Vector, Burlingame, CA, USA).

### 4.2. Gel Synthesis

**Figure 13:**
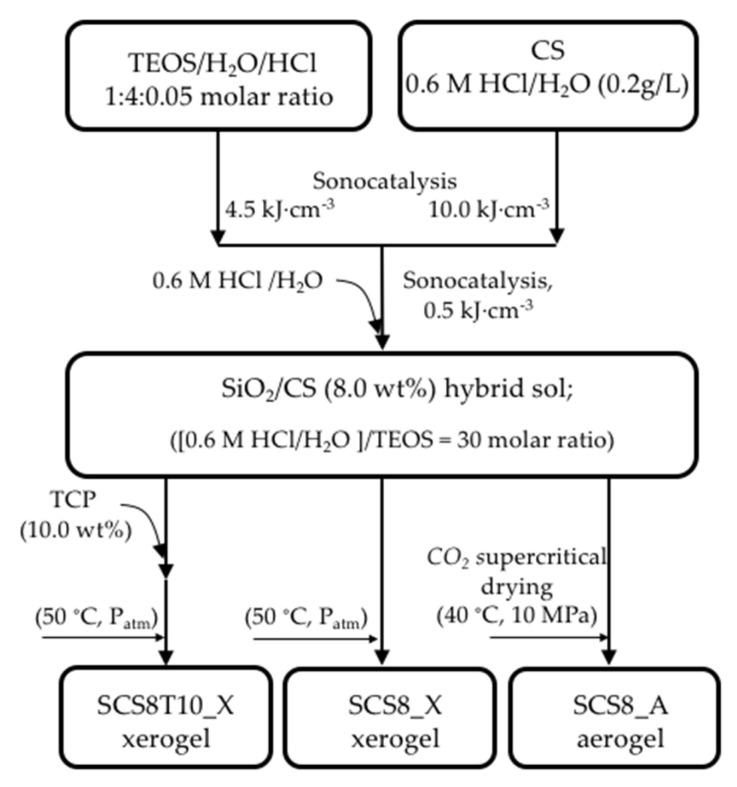
Process flow schematic for the production of SiO_2_/CS aerogels and SiO_2_/CS/TCP xerogels.

Chitosan–silica hybrid gels were synthesized using the sol-gel technique based on previously reported procedures [27,29] and schematized in Figure 13. First, silica sol was made by combining TEOS, water, and HCl in a molar ratio of 1:4:0.05, while exposed to 4.5 kJcm^−3^ of ultrasound energy in a glass reactor. A CS solution was prepared separately by adding 2.5 g of low molecular weight CS (50,000–190,000 Da) and 0.6M HCl to a 250 mL solution. A homogeneous mixture was obtained under the ultrasound exposure with an energy of 10 kJcm^−3^. The final solution was then obtained by combining both sols and adding an extra dose of ultrasound (0.5 kJcm^−3^), resulting in totally transparent solutions. All samples included 8% chitosan and the water/TEOS molar ratio was maintained at 30:1. TCP was then added to the TEOS/CS sol and completely dissolved to produce silica-8 wt.% CS, 10 wt.% TCP sols that were kept at 50 °C on a stove to gel for 24–48 h. The hybrid samples were then aged in an ethanol bath for 28 days to strengthen them and achieve the greatest shrinkage before drying. Finally, residual water in the hybrid hydrogel was removed after another week of soaking in ethanol with daily exchange. SCS8X and SCS8T10X xerogels were thus obtained by direct liquid evaporation at 50 °C, while supercritical drying in CO_2_ was employed to produce SCS8A aerogels. Samples were found in both cases as fracture-free monoliths with cylindrical geometry and well-differentiated volume contraction (see Figure 11).

According to our previous results (Perez-Moreno et al., 2020; Pérez-Moreno et al., 2021), the above synthetic procedure ensures that the resulting biomaterials exhibit high in vitro bioactivity responses as well as osteoconduction behavior in cell culture media [27,29]. In addition, the inclusion of 10 wt.% TCP has impact on the production of osteoinductive surfaces [27,71]. However, this TCP incorporation was unsuccessful for aerogels, because total TCP leaching was observed during the CO_2_ supercritical procedure.

### 4.3. Physical Characterization and Structural Properties

The mass of the samples was determined using a microbalance with an accuracy of 0.1 mg, and the sizes of monolithic specimens were measured by a Vernier caliper. Nitrogen physisorption tests (Micromeritics ASAP2010, Norcross, GA, USA) at 77 K with a pressure transducer resolution of 10^−4^ mm Hg were used to examine the textural characteristics of the hybrid aerogels. In order to conduct the study, specific surface area, pore volume, and pore size distribution were calculated using the BET and BJH standard models. The samples were degassed at 120 °C for 4 h before the tests.

### 4.4. Thermal Characterization

The thermal analysis of the materials was performed by thermogravimetric analysis (TGA) by measuring weight changes in the temperature range 50–900 °C in an air environment at a constant heating rate of 10 °C/min with a TGA Discovery instrument (TA Instruments, Ndw Castle, DE, USA).

### 4.5. Fourier Transform Infrared Spectroscopy

Fourier-transform infrared spectroscopy (FTIR) was employed to obtain information about the surface chemical structure of the samples. Experiments were recorded with a Bruker Alpha System Spectrophotometer (KBr wafer technique), using the same quantity of sample in all measurements. Signals were obtained in transmittance mode and the spectral range was set at 4000 to 500 cm^−1^ with a resolution of 4 cm^−1^.

### 4.6. Evaluation of the Bioactivity in SBF

Aerogel pellets measuring 5 mm in length and 8 mm in diameter were submerged in 20 mL of simulated body fluid (SBF) [61] in polyethylene flasks to study bioactivity. Hydroxyapatite (HAp) was then measured at the top after 28 days of soaking at 37 °C. The samples were meticulously removed from the buffer solution on a weekly basis, washed with Milli-Q water (Millipore Sigma, Burlington, MA, USA), and then dried once more at 50 °C and ambient pressure. The test was conducted with weekly fluid exchange. The surface morphologies of the SBF-treated samples were examined using an FEI Nova NanoSEM 450 (FEI, Morristown, NJ, USA) with a precision of 1.4 nm after various soaking times. Additionally, using a Bruker SDD-EDS analyzer, the Ca/P composition of the specimen surface was determined.

### 4.7. Cell Culture

Under sterile conditions, HOB cells were seeded onto the scaffolds. Once at optimal confluence, osteoblasts were extracted and counted to determine the optimal seeding density, and osteoblasts viability was determined using an automatic Luna^®^ cell counter (Invitrogen, San Diego, CA 92121 USA). The cell population doubling did not surpass ten. Both aerogels and xerogels were sterilized in a clinically standardized autoclave in accordance with the European standard DIN EN ISO 13,060 recommendations for class B autoclaves. After sterilization, the samples were placed in a laminar flow chamber in Mattek^®^ glass bottom wells under sterile conditions. Samples with a surface area of 1 square cm were used. A drop of 50 μL of cell suspension at a density of 15,000 HOB^®^ cells /cm^2^ was added to each sample and kept for 30 min, to avoid dispersion and ensure optimal cell attachment, in humid conditions and under incubation at 37 °C and 5% CO_2_. Following this, wells were refilled with OGM^®^ supplemented to a final concentration of 0.1 mL/mL of fetal calf serum and incubated during experimental times. Every two days, the media were replaced, and the degradation products were determined. The test groups were: SCS8A, SCS8X, SCS8T10X. Control groups consisted of HOB^®^ cells grown on glass.

### 4.8. Live/Dead Cell Assay

The cell viability and cytotoxicity of were assessed using a live/dead cell assay at experimental times of 24 h, 48 h, and 7 days. The samples were rinsed twice with PBS and exposed to calcein-AM (0.5 μL/mL) in PBS and ethidium homodimer-1 (EthD-1) (2 μL/mL) diluted in PBS. A confocal laser scanning microscope (CSLM) was employed to identify live and dead cells.

### 4.9. Cell Morphology and Spreading

Cell changes in morphology, alignment and spreading were assessed using a phase-contrast microscope prior to immunolabelling for fluorescence and confocal laser scanning microscopy. Both the fluorescence and confocal modes were combined, with the Nomarski mode for both material and cell imaging.

### 4.10. Actin Cytoskeletal Organization

Osteoblasts were immunolabeled with rhodamine-phalloidin and anti-vinculin antibodies after 48 h, 72 h, and 7 days of incubation. After washing with pH 7.4 prewarmed phosphate-buffered saline (PBS), samples were fixed with 3.7% paraformaldehyde at RT and permeabilized with 0.1% Triton x-100 after washing. Finally, preincubation with 1% bovine serum albumin in PBS for 20 min, after PBS rinsing, was performed in order to remove background prior to cell immunolabelling with rhodamine phalloidin and then rinsed with prewarmed PBS prior to mounting with Vectashield ^®^. At least five samples of each type were seeded and analyzed for each experiment. All experiments were repeated in triplicates unless otherwise stated. The test groups were: SCS8A, SCS8X, SCS8T10X. HOB cells grown on glass under the conditions described above were used as controls.

### 4.11. Confocal Examination

At least five samples were analyzed for each group using an Olympus confocal microscope to assess the influence of the surface on focal adhesion number and development, cytoskeletal organization, and cell morphology. At least 50 cells per sample were analyzed. Samples were exposed to the lowest laser power necessary to generate a fluorescent signal for a time interval not higher than 5 min to avoid photobleaching. Images were acquired at a resolution of 1024 × 1024, and processed.

### 4.12. Image Analysis

Sample images were collected as frames obtained at 40x magnification and processed using Image J software (NIH, http://rsb.info.nih.gov/ij (accessed on 26 June 2021). The perimeter, area, circularity, roundness, and aspect ratio were analyzed as shape variables. At least 40 regions of interest (ROIs) were measured for quantitative analysis. ROIs were selected under the following criteria: well-defined limits, clear identification of the nucleus, and absence of intersection with neighboring cells. Data were analyzed using SPSS and expressed as the mean ± standard deviation. Once normality and homoscedasticity were confirmed, the difference between the mean values was analyzed using one-way analysis of variance and the Brown–Forsythe and Games–Howell tests. Statistical significance was defined as *p* < 0.05.

## Figures and Tables

**Table 1:** Bulk density, volume shrinkage, and textural parameters calculated from N_2_-physisorption experiments of the xerogel (X) and aerogel (A) samples involved in this study.

Sample	ρ * (gcm^−3^)	Volume Shrinkage * (%)	S_BET_ ** (m^2^g^−1^)	V_p_ (cm^3^g^−1^)	Pore Size (nm)
SiO_2_A	0.19 ± 0.01	33.0 ± 2.3	978.2	4.1	16.9
SCS8A	0.18 ± 0.03	30.2 ± 3.1	857.7	3.9	17.3
SiO_2_X	0.61 ± 0.05	75.7 ± 2.6	807.5	1.0	4.7
SCS8X	0.49 ± 0.10	72.3 ± 4.2	821.1	1.1	5.0
SCS8T10X	0.54 ± 0.03	67.4 ± 2.4	733.6	1.5	7.5

* Errors indicate the standard deviation computed from three replicate measurements. ** Correlation coefficient for BET surface area measurements was higher than 0.9996 in all cases.

## Data Availability

Not applicable.
